# Embedding Permanent Watermarks in Synthetic Genes

**DOI:** 10.1371/journal.pone.0042465

**Published:** 2012-08-08

**Authors:** Michael Liss, Daniela Daubert, Kathrin Brunner, Kristina Kliche, Ulrich Hammes, Andreas Leiherer, Ralf Wagner

**Affiliations:** 1 Life Technologies/Geneart AG, Regensburg, Germany; 2 Department of Cell Biology and Plant Physiology, University of Regensburg, Regensburg, Germany; 3 Molecular Microbiology and Gene Therapy Unit, Institute of Medical Microbiology and Hygiene, University of Regensburg, Regensburg, Germany; Tata Institute of Fundamental Research, India

## Abstract

As synthetic biology advances, labeling of genes or organisms, like other high-value products, will become important not only to pinpoint their identity, origin, or spread, but also for intellectual property, classification, bio-security or legal reasons. Ideally information should be inseparably interlaced into expressed genes. We describe a method for embedding messages within open reading frames of synthetic genes by adapting steganographic algorithms typically used for watermarking digital media files. Text messages are first translated into a binary string, and then represented in the reading frame by synonymous codon choice. To aim for good expression of the labeled gene in its host as well as retain a high degree of codon assignment flexibility for gene optimization, codon usage tables of the target organism are taken into account. Preferably amino acids with 4 or 6 synonymous codons are used to comprise binary digits. Several different messages were embedded into open reading frames of T7 RNA polymerase, GFP, human EMG1 and HIV gag, variously optimized for bacterial, yeast, mammalian or plant expression, without affecting their protein expression or function. We also introduced Vigenère polyalphabetic substitution to cipher text messages, and developed an identifier as a key to deciphering codon usage ranking stored for a specific organism within a sequence of 35 nucleotides.

## Introduction

For millennia, mankind has employed the principle of consecutively selecting random mutations to breed desired phenotypes into crops, livestock, pets and microbes, relying on a trial-and-error approach. This was transformed when modern molecular biology enabled systematic genetic manipulation and redesign of novel strains and genetically modified organisms. Initially the focus was removing cross-species boundaries, rearranging natural genetic building blocks and introducing minor modifications into DNA sequences. Until recently almost all genetic templates originated from natural sources, limiting the range of possibilities.

The present dawn of synthetic biology opens up entirely new horizons in genetic engineering. It promises combining technical engineering approaches with biological sciences and informatics to predict, simulate, and construct novel pathways, genomes and organisms faster and more precisely. With the growing availability of low-cost *de novo* gene synthesis, synthetic biology not only allows unrestricted and flexible design of non-natural DNA sequences, but also adapting coding sequences to the genetic requirements of the chosen target organism. *In silico* design of an optimal coding sequence for a given protein using a distinct arrangement of alternative codons is known as “gene optimization”. Without altering the amino acid sequence, it is possible to enhance autologous and heterologous gene expression, adjust GC content, avoid sequence repetition, prevent silencing, and include/exclude defined sequence motifs [1].

Synthetic DNA also provides a digital medium for storing non-biological data. Several techniques have described using artificial DNA for hiding messages or direct storage of information [2]–[9]. For example, Clelland *et al.* [2] embedded information in a code consisting of a simple triplet code flanked by specific primers for its PCR amplification. The DNA is diluted with a large excess of spurious DNA and applied in microdots, such as the last period on a postcard. The recipient can extract the DNA, amplify the fragment carrying the message using the correct primer sequences, sequence it and read the information. In this case the sender and recipient have to agree on where to hide the DNA (period), how to amplify it (primer sequence), and how to encode it (triplet code).

A number of additional strategies for storing text messages in DNA have been described, although mostly by adding sequences that have no biological function but solely represent the stored text [2]–[4]. The recently created *Mycoplasma mycoides* with a fully synthetic genome contains four extra sequence elements of around 1 kb each containing non-coding sequences translated into English text also by an artificial triplet-to-character table [4]. The intention here was to prove the synthetic origin of the novel replicating genome and to testify the laboratory of origin. In addition to the encoded names of the scientists involved, and memorable quotations, was an Email address to send the decoded solution.

A more robust way to include or even hide a label or other information in an already information-containing product is steganography. As such, techniques to insert watermarks in digital media are quite common today. Images, for example, are a good medium for hiding messages by systematically modifying consecutive pixels. Usually the least significant bit of each pixel represents the consecutive bitstream of the watermark. This changes the color and/or brightness of each pixel by an insignificant amount and the presence of the watermark is not easily noticed. Furthermore, the watermark is inseparably interlaced into the file and cannot easily be removed.

Similarly, in contrast to adding extra non-coding sequences, direct watermarking of a gene adds the advantage that information is inseparably linked to the open reading frame (ORF), even when removed from its context. Ideally, a message needs to be integrated into the ORF without disturbing the product’s biological function. This strategy has already been applied by changing wildtype codons (binary 0) to alternative triplets (binary 1) which constrains the application of gene optimization to only those codons that encode binary 1. Codons representing binary 0 must, per definition, remain wildtype and are not amenable to optimization at all [5]–[8]. Another described approach is based on the systematic use of alphabetically sorted synonymous codons to embed a watermark by arithmetic coding. This inevitably determines every codon of the reading frame based solely on requirements of the watermark and not by means of decent biological performance [9]. Neither of these two systems is compatible with gene optimization since they leave little or no flexibility for adapting to genetic and biological requirements of chosen host organisms, or maintaining efficient gene expression.

The recent increase in more sophisticated genetically modified organisms and wide use of synthetic genes in molecular engineering prompted us to evaluate the biological applicability of steganographic storage of watermark messages within protein reading frames, without compromising gene optimization requirements or expression.

Here, we describe embedding various text messages into the ORFs of various genes already optimized for expression in bacteria, yeast, plants or humans. In all cases gene expression was comparable to the parental version, as was the watermarked protein function when analyzed for cellular localization or enzymatic activity.

## Results

### Embedding Watermarks into Coding Genes

**Figure 1:**
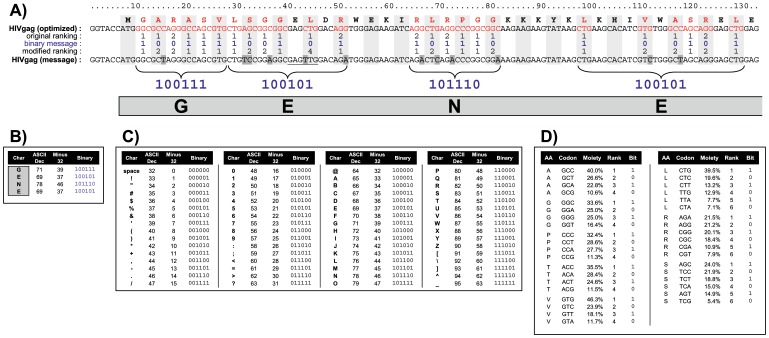
Embedding a watermark into a functional gene. A) The 5′ end of HIV gag ORF optimized for *Homo sapiens*, HIVgag (optimized) with its amino acid and nucleotide sequence, and original codon ranking. The modified codon ranking and altered nucleotide sequence of HIVgag (message) shown below give the desired binary message in blue, spelling “GENE”. B) The ASCII symbols spelling “GENE” convert to the binary digits 100111, 100101, 101110, 100101, based on C) the modified ASCII table. A total of 64 typographic characters (Char) were chosen from the print characters 32 to 95 of the standard ASCII decimal code (ASCII Dec). Subtracting 32 from each value gave numbers ranging from 0 to 63 (Minus 32), which were converted into a 6-bit binary code (Binary). D) The sorted human codon usage table was used to incorporate this bitstream into the modified HIVgag (message) sequence depicted above. Only amino acids with ≥4 alternative codons were changed (red letters in HIVgag sequence at the top). Binary 1 represents codons ranking 1, 3 or 5 (odd); binary 0 is for codons ranking 2, 4 or 6 (even). To secure binary 0 at nucleotide position 43 the leucine codon ranking 4 was chosen since the 2^nd^ best codon would have created an undesirable *SacI* restriction site (GAGCTC). Embedding the four-letter text message required 12 silent substitutions (shaded grey in A) in the watermarked DNA sequence.

The approach we developed to insert a watermark into a functional gene first requires translating the plain watermark text into a binary format. Here, we used the common ASCII code, but to save space and store 25% more text in the reading frames, we reduced the regular 8-bit ASCII code to a 6-bit word size by subtracting 32 from each letter value. This allows coding for 2^6^ = 64 typographic characters, starting from number 32 (space) through to 95 (underscore) (Fig. 1C). For example, the four-letter message “GENE” is translated into the 24-digit string of bits “100111 100101 101110 100101” (Fig. 1B).

Next, this bitstream must be represented in the coding gene without affecting the sequence of the translated protein. Clearly, the degeneracy of the genetic code is ideally suited for this purpose; however care must be taken not to restrict the system in a way that interferes with the intended biological performance of the gene. Not all possible codon combinations for a given protein perform equally well. Indeed, a prominent rationale for gene optimization is to deliberately influence a gene’s behavior, normally to secure or increase its expression [11], [12].

A central parameter for optimizing species-specific attributes of genes is the codon usage table of the host organism, in other words a numerical representation of the overall frequencies of synonymous codons in the organism’s genome (Fig. 1D & Table S1). Although different strategies can be used for gene optimization, one generally focuses on the more frequent codons of the target species when adapting a synthetic gene *in silico* [1]. We therefore decided to use the frequency table of the respective host organism as the key for embedding a watermark bitstream into the reading frame of a synthetic gene. In our case, synonymous codons are sorted according to their relative frequencies rounded to one decimal place. If two synonymous codons share the same frequency, these are sorted alphabetically (e.g. GGA/GGG for glycine; Fig. 1D). The resulting species-specific ranking of synonymous codons can be used to selectively symbolize binary data as well as the protein-coding information of a gene.

In general, we designed the ORF of a watermarked gene from ATG to stop by representing the 1st, 3rd or 5th synonymous codon (odd ranking) for a particular amino acid by binary 1, and binary 0 for the 2nd, 4th or 6th codon (even ranking). This key for the relationship between the gene and the watermark bitstream is host-specific and allows gene optimization to be compatible with influencing the desired performance of the synthetic gene in the chosen system. For this reason we also found it best to confine data storage to only those amino acids encoded by four or six alternative codons (AGPTVLRS; Fig. 1D), hence retaining a high degree of codon assignment flexibility by having the choice between at least two codons in the design process. This provides enough flexibility to maximize gene optimization and minimize undesirable DNA sequences. For example, to watermark the HIV gag ORF in Figure 1 the watermarking algorithm selected the 1st and 2nd best codon in most cases, but avoided creating an undesirable *SacI* restriction site by choosing the 4th ranking leucine codon to encode binary 0.

### Reading Watermarks in Labeled Coding Genes

To extract the watermark from a labeled gene, the process described above is reversed. Again, the key for allocating codons to binary data is the sorted codon usage table of the organism in question (although it can also be an artificial table as described under: Codon usage table attachment). Only codons for the amino acids A, G, P, T, V, L, R, and S are taken into account (in red in Fig. 1A). The deduced binary string is then segmented in 6-bit words and converted to the ASCII characters by adding 32 (Fig. 1C).

### HIVgag Expression in HEK293 Cells

**Figure 2:**
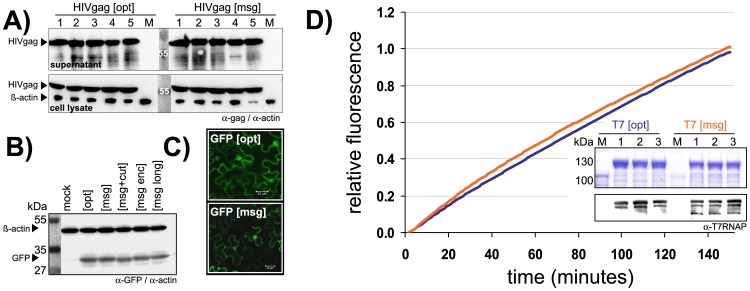
Protein expression and functionality. A) Western blots of HIV gag protein without [opt] and with [msg] message expressed in HEK293 cells. Equal amounts of protein from 5 independent transfections were analyzed from cell supernatants (top panels) and cell lysates (bottom panels), with actin detection serving as a control. B) Western blot of HEK293 lysates expressing GFP from optimized genes without [opt] and with [msg] embedded watermarks, including the appended human codon ranking sequence [msg+cut] (see Fig. 3), an encrypted watermark [msg enc] (see Table 2) and with a longer embedded message [msg long], also employing amino acids with 2 or 3 alternative codons (CDEFHIKNQY). Protein expression was quantified by densitometry. Results are derived from five independent experiments. C) Fluorescence microscopy images of GFP transfected into tobacco leaves show no visible differences in cellular location and only little variation in abundance. D) GST-T7 RNA polymerase [opt] and [msg] expressed in triplicates in *E. coli* was analyzed by SDS-PAGE and Coomassie staining (top panel) or Western blotting using a specific T7 RNA polymerase antibody (α-T7RNAP; lower panel). Equal amounts of purified T7 RNA polymerase were used for *in vitro* transcription. Synthesized RNA detected with a molecular beacon in real-time directly revealed almost identical RNA polymerase activities mediated by T7 [opt] (blue line) and T7 [msg] (orange line).

To test whether watermarking affects protein expression or functionality, we first analyzed HIV gag (pr55^gag^ from HIV1) optimized for expression in human cells. The Gag gene was modified to encode the message “GENE DESIGNED BY MARCUS GRAF/GENEART 2008.”. After transiently transfecting both the optimized [opt] and watermarked [msg] constructs into human embryonic HEK293 kidney cells, protein amounts expressed in cell lysates were indistinguishable (Fig. 2A). Although the HIV structural protein Gag is not secreted, budding results in shedding of virus-like particles containing Gag protein into the supernatant, where protein levels detected by ELISA were also the same for both constructs (3% deviation; data not shown).

### GFP Expression in HEK293 Cells

We tested four different GFP gene variants (Table 1): GFP [msg] codes for the text “AEQUOREA VICTORIA.”; GFP [enc] carries the encrypted message “4JT′T&8F#(NWGTU[FB”, and the password “Secret” reveals the original message “AEQUOREA VICTORIA.” (see: Vigenère polyalphabetic substitution). The construct GFP [msg+cut] is identical to GFP [msg] followed by a 35 bp non-coding sequence added immediately after the stop codon of the reading frame (see: Codon usage table attachment). In the construct GFP [long] the codons for amino acids (CDEFHIKNQY) with 2 or 3 alternative codons were also used for watermarking, making use of the whole coding space to carry the longer message “GREEN FLUORESCENT PROTEIN GENEART 2008”.

**Table 1:** Summary of the genes tested. Wildtype gene sequences optimized for expression in different organisms^1^ were used for embedding various messages.

Protein	Protein Length	Optimization [Encoding]	Message	Message Length		CAI		GC		Rel. Expression (msg : opt)	Assay System
					Δ opt : msg	Opt	msg	opt	msg		
GST-T7RNAP	1134 aa	*E. coli* [msg]	GENEART AG, GERMANY/THE GENE OF YOURCHOICE/MARCH 19TH 2008/WAGNER & LISS….	83 char 498 bit	351 bp 10%	0.90	0.85	50%	47%	1.05±0.21 0.80	*E. coli*
GFP	239 aa	*S. cerevisiae* [msg]	AEQUOREA VICTORIA.	18 char 108 bit	52 bp 7%	0.95	0.86	35%	34%	0.87±0.20 0.37	*S. cerevisiae*
GFP	239 aa	*A. thaliana* [msg]	AEQUOREA VICTORIA.	18 char 108 bit	69 bp 10%	0.92	0.89	44%	44%	0.94±0.39 0.67 1.03±0.22 0.91	*N. benthamiana in vitro* - wheat
HIVgag	513 aa	*H. sapiens* [msg]	GENE DESIGNED BY MARCUS GRAF/GENEART 2008.	45 char 270 bit	167 bp 11%	0.99	0.90	65%	59%	1.03±0.05 0.40	HEK 293
EMG1	252 aa	*H. sapiens* [msg]	GENEART AG PAT US1234567	24 char 144 bit	92 bp 12%	0.79	0.87	64%	59%	0.92±0.12 0.35	HEK 293
EMG1	252 aa	*H. sapiens*[msg enc]	:JQWF&G%DY%$4Y#′XE%87G;K Pwd “Secret” →GENEART AG PAT US1234567	24 char 144 bit	93 bp 12%	0.79	0.87	64%	59%	0.67±0.27 0.19	HEK 293
GFP	239 aa	*H. sapiens* [msg]	AEQUOREA VICTORIA.	18 char 108 bit	63 bp 9%	0.97	0.91	59%	57%	1.12±0.31 0.61 1.04±0.25 0.89	HEK 293 *in vitro* - rabbit
GFP	239 aa	*H. sapiens*[msg enc]	4JT′T&8F#(NWGTU[FB Pwd “Secret” →AEQUOREA VICTORIA.	18 char 108 bit	68 bp 9%	0.97	0.90	59%	55%	1.00±0.22 0.91 1.07±0.37 0.88	HEK 293 *in vitro* - rabbit
GFP	239 aa	*H. sapiens*[msg + cut]	AEQUOREA VICTORIA.	18 char 108 bit	63 bp 9%	0.97	0.91	59%	57%	0.92±0.07 0.20 0.91±0.26 0.54	HEK 293 *in vitro* - rabbit
GFP	239 aa	*H. sapiens*[msg long]	GREEN FLUORESCENT PROTEIN GENEART 2008	38 char 228 bit	126 bp 18%	0.97	0.83	59%	48%	0.81±0.17 0.23 0.71±0.43 0.33	HEK 293 *in vitro* - rabbit

[msg]  =  Only codons for amino acids with ≥4 alternative codons (AGPTVLRS) were used for data storage. [msg enc]  =  Text message was keyed with the password “Secret” using the polyalphabetic Vigenère cipher. [msg + cut]  =  the codon usage table for *Homo sapiens*, used for encryption, was added in a 35 bp sequence after the stop codons of the ORF. [msg long]  =  additional codons for amino acids (CDEFHIKNQY) were used for message embedding, doubling the capacity of the container gene. Δ opt:msg  =  total number and percentage of altered nucleotides resulting from message deposition. CAI and GC  =  codon adaptation index and GC content of optimized and watermarked genes. Rel. Expression  =  mean relative expression ratio between watermarked and optimized genes ± standard deviation and students t-test p-value. Assay systems included expressing constructs *in vivo* in *E. coli* BL 21, *S. cerevisiae* strain AH109, human HEK293 cells, and *in vitro* with wheat germ and rabbit reticulocyte lysates; protein expression was determined by Western blots and ELISA.

Expression of GFP constructs [msg], [msg+cut], and [enc] was very similar, differing by 12%, 8%, and 0% from the optimized gene, as quantified by densitometry (Fig. 2B). In contrast, GFP [long] showed slightly decreased expression, reaching 0.81-fold of the optimized gene. This is not surprising since the size of the encoded message necessitates 126 nucleotide substitutions. Since a number of codons were constrained by the watermark rather than gene optimization, this results in a significant change in the codon adaptation index and GC content (Table 1). All the GFP constructs were also tested in *in vitro* translation with the rabbit reticulocyte lysate system, giving similar results (data not shown).

### GFP Expression in Yeast and Plants

Constructs encoding GFP optimized for expression in yeast, were modified to give the watermarked version containing 52 silent substitutions encoding the text “AEQUOREA VICTORIA.”. Whole cell lysates were analyzed by Western blotting using a specific GFP antibody. Compared to the optimized gene the watermarked GFP showed 0.87-fold expression on average, although this difference is not statistically significant (p-value 0.37; Table 1). GFP optimized for expression in dicotyledons (*A. thaliana*) with or without the message “AEQUOREA VICTORIA.” expressed in tobacco leaves were visualized by *in situ* fluorescence microscopy (Fig. 2C), and quantified by Western blotting (6% deviation; data not shown). *In vitro* translation of the GFP constructs using a wheat germ lysate system also revealed no difference in expression (3% deviation; results not shown).

### GST-T7 RNA Polymerase Expression in *E. coli*

GST-T7 RNA polymerase from bacteriophage T7, optimized for expression in *E. coli*, contained an N-terminal GST-tag. Genes without [opt] and with the watermark [msg] encoding the message “GENEART AG, GERMANY/THE GENE OF YOUR CHOICE/MARCH 19TH 2008/WAGNER & LISS….” were expressed in *E. coli*, and whole cell lysates were analyzed by Western blotting (Fig. 2D). Expression of the watermarked gene was comparable to the optimized gene (5% deviation; Table 1). In parallel, the protein was affinity purified via its GST-tag using GSH-agarose and analyzed by SDS-PAGE (Fig. 2D). Equal amounts of this purified T7 RNA polymerase were used in an *in vitro* transcription assay detecting synthesized RNA with a specific fluorescent probe (molecular beacon) in a real-time cycler. No differences in RNA transcription kinetics or final amounts of synthesized RNA were observed, confirming that the T7 RNA polymerases expressed from the optimized and watermarked genes were functionally identical (Fig. 2D).

### EMG1 Expression in HEK293 Cells

The nucleolar protein homologue EMG1, optimized for expression in human cells, was tested with two variants (Table 1): His-tagged EMG1 [msg] containing the coded message “GENEART AG PAT US1234567” and a second His-tagged version, EMG1 [enc] encoding an encrypted message using the Vigenère cypher method that can be decoded with the password “Secret” (see: Vigenère polyalphabetic substitution). After transient transfection of HEK293 cells, protein expression was quantified by Western blotting using an anti-His antibody against the N-terminal penta His-tag. Expression of EMG1 [opt] and EMG1 [msg] was almost equivalent (8% deviation). In contrast, EMG1 [enc] expression was only 0.67-fold compared to the parental optimized construct (Table 2). Possible reasons for this 33% deviation are as yet unknown.

**Table 2:** Vigenère polyalphabetic substitution.

Msg	Ascii	Pwd	Ascii	Msg+Pwd	→ Mod 64	→ +32	Enc	Enc-Pwd	→ Mod 64	→ +32	Msg
**A**	65	**S**	83	148	20	52	**4**	−31	33	65	**A**
**E**	69	**e**	101	170	42	74	**J**	−27	37	69	**E**
**Q**	81	**c**	99	180	52	84	**T**	−15	49	81	**Q**
**U**	85	**r**	114	199	7	39	**′**	−75	53	85	**U**
**O**	79	**e**	101	180	52	84	**T**	−17	47	79	**O**
**R**	82	**t**	116	198	6	38	**&**	−78	50	82	**R**
**E**	69	**S**	83	152	24	56	**8**	−27	37	69	**E**
**A**	65	**e**	101	166	38	70	**F**	−31	33	65	**A**
	32	**c**	99	131	3	35	**#**	−64	0	32	
**V**	86	**r**	114	200	8	40	**(**	−74	54	86	**V**
**I**	73	**e**	101	174	46	78	**N**	−23	41	73	**I**
**C**	67	**t**	116	183	55	87	**W**	−29	35	67	**C**
**T**	84	**S**	83	167	39	71	**G**	−12	52	84	**T**
**O**	79	**e**	101	180	52	84	**T**	−17	47	79	**O**
**R**	82	**c**	99	181	53	85	**U**	−14	50	82	**R**
**I**	73	**r**	114	187	59	91	**[**	−23	41	73	**I**
**A**	65	**e**	101	166	38	70	**F**	−31	33	65	**A**
**.**	46	**t**	116	162	34	66	**B**	−50	14	46	**.**

Polyalphabetic substitution cypher of the plain message (Msg) “AEQUOREA VICTORIA.” with the key password (Pwd) “Secret” leading to the encrypted text (Enc) “4JT′T&8F#(NWGTU[FB” as found in GFP [enc], one of the constructs analyzed in Fig. 2C.

### Codon Usage Table Attachment

**Figure 3:**
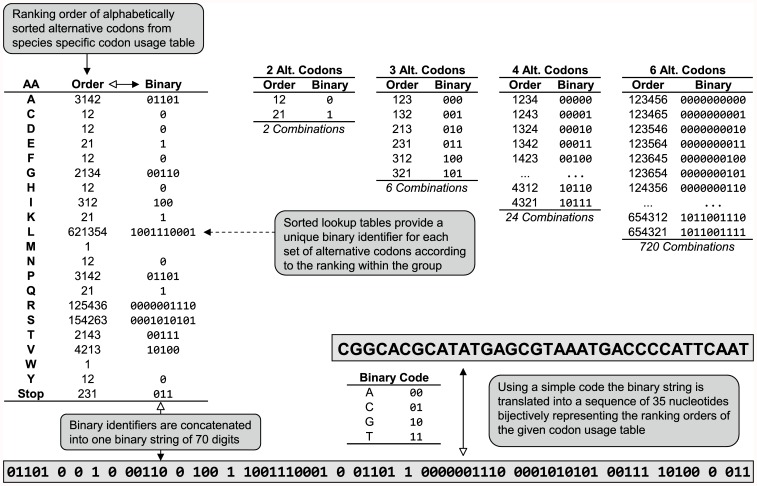
Codon usage table attachment. Procedure for storing the key to any possible codon usage table ranking order within a string of 35 nt (▴) and *vice versa* (Δ). In this example the sequential arrangement of the human codon usage is specified in the table columns “AA” and “Order”. For example, the most frequently used alanine codon in the human genome is GCC, followed by GCT, GCA and the least frequent GCG. Applying this sequential frequency arrangement to the alphabetically ordered codons results in GCA(3), GCC(1), GCG(4), GCT(2) or - in short - 3142. In total, there are 24 possible combinations of 4 alternative codons (#0 = 1234 … #23 = 4321) and the showcase order 3142 is at position #13 in this list. Thus, from the lookup table for four alternative codons (4 Alt. Codons), this order can be represented by the binary identifier 01101 ( = 13). When performed for each amino acid, a binary string of 70 digits defines the frequency arrangement of all 64 codons for that specific codon usage table, here listed for *H. sapiens*. A simple binary-to-nucleotide translation table (A = 00, C = 01, G = 10, T = 11) can then represent this binary string in a sequence of 35 nucleotides.

The codon usage table (CUT) together with the moieties and rankings of alternative codons provides the key to reading the watermark message within coding genes (Fig. 1D & Fig. 3). Although today a CUT is not subject to change for most fully sequenced species of interest, it can be optionally stored together with the gene with little extra effort or costs. The construct GFP *H. sapiens* [msg+cut] (Table 1) contains the watermark “AEQUOREA VICTORIA.” within the open reading frame and adds the non-coding nucleotide string CGGCACGCATATGAGCGTAAATGACCCCATTCAAT just downstream of the stop codon (Fig. 3). These 35 bp permanently log the ranking of all 64 codons according to the human CUT (Table S1 in column *H. sapiens*) and therefore provide the key to decoding the watermark. A similar strategy with a different CUT can be applied for decoding a watermark in cases where the codon usage table of the host organism is unknown.

### Vigenère Polyalphabetic Substitution

In two constructs (EMG1 *H. sapiens* [msg enc] and GFP *H. sapiens* [msg enc]; Table 1) the plain text message was encrypted with the simple but recognized Vigenère polyalphabetic substitution cipher prior to embedding in the ORF (Table 2). In the latter example the ASCII values of the plain message (Msg) “AEQUOREA VICTORIA.” are added to the ASCII values of the processing password (Pwd) “Secret” followed by a modulo 64 operation (remainder of division by 64) to reduce the word size from 8 to 6 bit. Subsequently, adding 32 adjusts these numbers back to regular ASCII values between 32 and 95. The ASCII characters of these integers yield the encrypted (Enc) text “4JT′T&8F#(NWGTU[FB” and are compatible for watermarking according to the allocation table in Figure 1C. To decode a Vigenère encrypted watermark, the ASCII digits of the password are subtracted from those of the watermark and then subjected to modulo 64 and adjusted back to ASCII format by adding 32.

### Watermark Stability in HIV Gag

**Figure 4:**
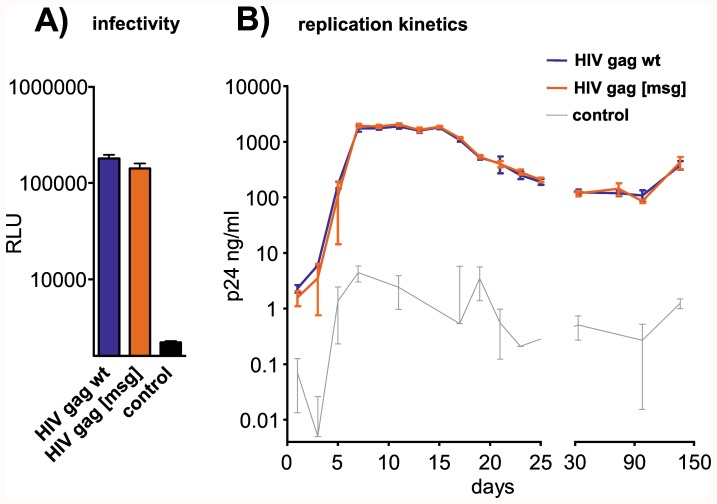
Influence of gene labeling in HIV-1 gag on viral infectivity and replication. A) Viral infectivity was assessed after transiently infecting HEK293T cells with the indicated viral plasmids, harvesting cells 72 h post transfection and using equal amounts of capsid-normalized virus particles to infect CD4-positive TZM-bl indicator cells. Luciferase activity (RLU) was measured in cells lysed 48 h post infection. The control represents uninfected cells. Error bars indicate the standard deviations from quadruplet infection. B) To monitor and maintain viral replication over a period of 20 weeks, CEM cells were infected in duplicate with virus-containing supernatants. Capsid protein (p24) amounts in culture supernatants were quantified by ELISA at the indicated time points. The control represents supernatant from an uninfected cell culture. Error bars indicate standard deviations from duplicate infections.

Although the overall expression levels of watermarked optimized genes were comparable to the parental optimized genes in most cases, natural mutation rates may limit their applicability. To determine the stability of such watermarks, we applied gene labeling to a fast mutating organism, HIV. As before, we used watermarking to introduce the message “[REGENSBURG]” into the 3′ part of the gag reading frame (NL4-3), using the watermarking key based on the human codon usage table (Fig. 1D). However, since complete humanization of lentiviral genes seriously disturbs viral gene regulation and replication [17], we used wildtype gag sequence for watermarking instead of an optimized gene. The watermarked version contains 37 silent nucleotide substitutions in a stretch of 168 codons. In agreement with the results above, HIV infectivity and thus replication was not notably affected by introducing the message. More importantly, no mutation or reversion was detected in the watermarked gag sequence after 136 days of replication in non-permissive CEM cells (Fig. 4).

## Discussion

A variety of cryptographic and steganographic techniques have been developed in the past, using increasingly sophisticated algorithms to cipher non-biological information in DNA. Such watermarks were usually inserted as extra non-coding sequences [2], [3], [4], although several groups have stored text messages within the protein coding sequence itself [5]–[9]. For the first time, we have inserted text messages into the protein coding sequences (ORFs) of several proteins expressed in various organisms and *in vitro* expression systems, without compromising gene expression, message stability, or protein function.

We analyzed four different genes (T7 RNA polymerase, GFP, human EMG1 and HIV gag) ranging across various phylogenic expression systems (*in vivo*: bacteria, yeast, plants, and human cells; *in vitro*: wheat germ and rabbit reticulocyte lysates). In two examples, the encoded text was further encrypted by Vigenère polyalphabetic substitution prior to embedding into the ORF (Table 2). Recombinant protein expression analyzed by Western blotting, ELISA or fluorescence microscopy revealed no significant differences between original optimized, or substituted watermarked constructs in almost all cases. Moreover, no detectable loss of protein function was confirmed for purified GST-T7 RNA polymerase using real-time *in vitro* transcription assays (Fig. 2D). The reason for a slightly higher degree of degradation of this protein when expressed from the watermarked gene remains speculative. It may be caused by translational pausing effects and divergent folding kinetics or a somewhat longer doubling time of the watermarked culture after induction (47 min versus 43 min).

To retain a high degree of codon assignment choice and gene optimization flexibility, we found it best to confine data storage to amino acids encoded by four or six alternative codons (AGPTVLRS). On average, about 50% of a protein comprises these amino acids, thus a gene encoding a protein of 300 amino acids can store about 150 bits or 25 characters (6 bits per character). To double data capacity, one could include amino acids with 2 or 3 synonymous codons (CDEFHIKNQY). Then reading 5′ to 3′, each codon, except Met or Trp, can be used to store one bit. However, this is at the cost of gene optimization flexibility, and results in significantly changing the codon adaptation index and GC content. The potential adverse consequence is demonstrated by GFP expression in HEK293 cells, where the [msg long] version was expressed at lower levels due to the 126 nucleotide substitutions needed to accommodate the longer message.

DNA sequences containing a message have previously been introduced into living organisms without disrupting their functions. Wong *et al.* used a vector that integrated into the genome of *Deinococcus radiodurans*, a microorganism surviving extreme conditions, to add extra DNA translating into “AND THE OCEANS ARE WIDE” using an artificial triplet code [3]. Gibson *et al.* also employed non-protein coding DNA and a direct triplet-to-character code to label the synthetic genome of *Mycoplasma mycoides* with four 1 kb blocks at defined positions [4]. Arita *et al.* developed a steganographic algorithm based on the degenerative genetic code, introducing point mutations in redundant codons. They encoded “KEIO” into the *Bacillus subtilis* ftsZ gene, essential for cell division, and demonstrated that the modified codon sequences did not affect cell division, colony morphology, growth rate or sporulation frequency. To extract their encoded message, one must know the wildtype sequence to decide which codons are not modified (binary 0) or diverge (binary 1) from the original sequence [5].

In contrast, knowing the wildtype or optimized sequence is not required to decypher the message in our watermarked constructs. The only necessary information, or “key” is the codon usage table (CUT) of the host organism. Today, most strains and species used in genetic engineering are fully sequenced, thus their codon usage table data is not subject to further change and is publicly available. However, the option of storing the key to any possible CUT in a string of 35 nucleotides (Fig. 3) might be useful if little sequence data is available, or the codon usage data is inaccurate for a particular organism of choice. Alternatively, one might want to use a completely artificial CUT intended for using the watermarked gene in different organisms or expression systems.

One obstacle to introducing messages into the DNA of living organisms is their ability to evolve over time. Mutations within the integrated DNA sequence can be corrected using several mutation correction codes to keep the information intact [6]. However, our watermark in the gag reading frame of the fast mutating human immunodeficiency virus remained intact after 136 days of virus replication, suggesting no intrinsic instability leading to selected mutations or reversions caused by introducing the silent nucleotide message substitutions.

If the aim of an artificial text message in a living organisms is not to pass on information but to label the gene or organism it is mandatory that the performance of gene expression is not impaired by the necessary silent point mutations. In Vam7, a protein from yeast involved in sporulation, it has been shown that hidden information or a DNA watermark does not affect mRNA translation and the resulting protein is functionally intact [6], [7]. The applicability of DNA watermarks was also shown *in silico* for sexually reproducing diploid organisms, which represent a special challenge, since additional recombination and crossover events can destroy integrated watermarks. A coupled Y-chromosomal/mitochondrial DNA watermarking procedure was identified as the most appropriate for diploid organisms [8].

The possibility of introducing messages into non-coding regions, such as promoters, was also recently tested [10]. Since in one case a promoter lost its function due to the introduced message, integrating watermark sequences into regulatory regions cannot be generally recommended.

Our results illustrate a strategy for embedding gene watermarking that is stable and compatible with expressing optimized synthetic genes in bacteria, yeast, animals and plants. By exploiting only those amino acids with 4 or 6 alternative codons, the degree of flexibility is high enough to allow for suitable gene optimization, and importantly, avoid undesired DNA motifs, such as restriction sites and GC content.

Besides the simple transport of extra information, one reason to interlace messages in a DNA sequence is to authenticate genetically modified organisms. Apparent or hidden branding of genes and organisms may become an important feature in synthetic biology to identify the manufacturer or patentee, label intellectual property, or provide batch numbers, company or product names, dates or warning notices. Gene labeling will also have value in biosecurity and biosafety appliances and allows for consistent traceability in particular if its application is mandatory for release approvals. Live vaccine development may benefit from unique and stable watermarks that clearly discriminate between vaccination cases or natural infection. Furthermore, many genetic engineering products have a significant commercial value and it is obvious that branding or tagging of such products is as important as for other industrial goods.

Directly incorporating information into the open reading frame of a functional gene has many advantages. The gene itself is the carrier, using the versatility of the degenerated genetic code. The method described here enables the labeling of single genes, viruses, microorganisms, plants or animals. Since the information is inseparably associated with the modified gene, it is inconspicuous, and more importantly, indelible even if the gene is transferred into another organism.

## Materials and Methods

### Construct Design and Gene Synthesis

The coding regions of gene sequences retrieved from the NCBI GeneEntrez database were optimized using the GeneOptimizer® expert software system (Geneart AG) as described before [1], [11], [12]. Bioinformatic embedding of text messages into the optimized genes is described in Results. For text-to-binary conversion the common ASCII code was reduced to a word size of 6 bit by subtracting 32 from each letter value (Fig. 1C). This covers the 64 ASCII characters 32 to 95:

space!”#$%&′()*+,−./0123456789:;< = >?@ABCDEFGHIJKLMNOPQRSTUVWXYZ[\]?_.

Sequences of all optimized and watermarked genes are reproduced as supplementary data (File S1). Alignments of protein, optimized gene, watermarked sequence, binary and plain text message for *Homo sapiens*-optimized GFP [msg] and GFP [msg long] are illustrated in Figure S1. Following *in silico* design all optimized genes were assembled from synthetic oligonucleotides (*de novo* gene synthesis), cloned in appropriate expression vectors and verified by sequencing prior to further processes.

### Protein Expression in E. coli

Constructs for expression in *E. coli* were cloned into pEG-His1 (Mobitec) and transformed into *E. coli* BL21. Three independent colonies were inoculated into 10 ml Luria-Bertani (LB) broth containing ampicillin (50 µg/ml) and grown overnight with shaking at 250 rpm. 1 ml of the overnight cultures was then used to inoculate 10 ml of LB broth containing ampicillin (50 µg/ml). Cultures were grown to an OD_600_ of 0.6 and expression was induced by adding 1 mM IPTG. Cells were harvested by centrifugation 3 h after induction. The cell pellet was resuspended in 100 µl of loading buffer, sonicated briefly and 10 µl were used for Western blotting.

### Protein Expression in HEK293 Cells

Constructs for expression in HEK293 cells were cloned into pTriEx1.1 (Novagen). The day before transfection, adherent HEK293 cells were seeded in 6-well plates with 750,000 cells per well. Before transfection the medium was replaced with 1 ml of OptiPro (Invitrogen) supplemented with 4 mM glutamine and 10 mM HEPES. For transfection in one well 2 µg DNA were diluted in 100 µl of OptiPro and 1 µl polyethylenimine (PEI, 1 µg/ml in H_2_O, Polyplus) was added. The mixture was incubated for 10 min at room temperature and added to the cells. 6–12 h post-transfection the medium was replaced with normal growth medium. On day 3 after transfection cells were harvested. For HIV capsid protein (p24) the supernatant was transfused and clarified from cell debris by centrifugation (5 min at 12,000 g). To quantify GFP and EMG1 expression cells were washed with PBS and resuspended in 500 µl lysis buffer. Further lysis and DNA degradation was performed using sonification. Total protein was quantified using DC-Protein assay (Biorad) according to the manufactures instructions. Equal amounts of total protein were used for Western blotting and p24 ELISA.

### Protein Expression in S. Cerevisiae

Yeast constructs were cloned into a pRS423 derivative containing an ADH1 promoter and LEU2 terminator for expression. Transformation of yeast strain AH109 was performed as described [15]. For gene expression three independent colonies were inoculated into 10 ml YPD medium and grown overnight with shaking at 190 rpm. Overnight cultures were then used to inoculate 10 ml of YPD medium at an OD_600_ of 0.5. Cells were harvested after 5 h growth at 30°C and shaking at 190 rpm. Equal amounts of cells were resuspended in 50 µl Tris·HCl pH 6.8 containing 2% (w/v) SDS. About 50 mg of glass beads were added to the cells and vortexed for 1 min. The suspension was incubated at 95°C for 10 min and vortexed again. Glass beads and cell debris were removed by centrifugation and 5 µl of the supernatant was used for Western blotting.

### Protein Expression in Tobacco

GFP variants were expressed by infiltration into *Nicotiana benthamiana* leaves as described [13]. Confocal UV microscopy was performed two days after infiltration on a Zeiss LSM510. Proteins were extracted from infiltration areas two days after infiltration as described [14] without GTP in the grinding buffer.

### *In vitro* Protein Expression

Constructs were cloned into pTriEx1.1 (Novagen) and expressed *in vitro* using the following systems according to the manufacturer’s protocol: TNT® T7 Coupled Reticulocyte Lysate System, and TNT® T7 Coupled Wheat Germ Extract System (Promega). Briefly, 0.2 µg of plasmid DNA were used for 10 µl reactions. After incubation at 30°C for 4 h, 5 µl of the reactions were used for Western blotting. Reactions containing wheat germ extract were used directly, whereas reactions containing rabbit reticulocyte lysate were precipitated with acetone prior analysis.

### Quantification of Protein Expression

Recombinant protein-specific bands on Western blots were visualized using BM Chemiluminescence substrate (Roche) and detected using the Chemilux ECL Imaging System (Gel iX Imager, Intas). Corresponding constructs were analyzed on the same Western blot. Signals were quantified by densitometry using the software scion image (Scion Corporation). Lysates from cells transformed or transfected with an empty expression construct, or *in vitro* reactions lacking a DNA template served as negative controls.

### Purification and Activity Assay of GST-T7 RNA Polymerase

Expression constructs were transformed into *E. coli* BL21. Cultures were grown at 30°C and harvested by centrifugation 4 h after induction. Bacteria were resuspended in 10 ml disruption buffer (PBS containing 0.1% TX-100 and protease inhibitor cocktail). The suspension was sonicated for 1 min and the lysate clarified by centrifugation. Glutathione-agarose beads (100 µl, Sigma) were added to the same amount of lysate and incubated for 10 min at 4°C. Beads were washed twice with 500 µl PBS and then twice with T7 buffer (10 mM Tris·HCl pH 8.0, 100 mM NaCl, 0.1% TX–100, 10 mM DTT). Bound protein was eluted with 50 µl of T7 buffer containing 10 mM reduced GSH (Roth). To test for enzyme activity, equal amounts of protein were used for *in vitro* transcription of the *Renilla* luciferase gene under the control of a T7 promoter. One reaction contained 1× RNA polymerase reaction buffer (NEB), 5 mM NTPs, 5 mM MgCl_2_, 25 ng DNA template (*Renilla* luciferase), 10 ng purified GST-T7 RNA polymerase and 4 µM molecular beacon for the specific detection of luciferase RNA. Fluorescence was detected in a real-time cycler (MiniOpticon, BioRad) at a constant temperature of 37°C for 150 min.

### HIV-1 Infectivity and Long-term Replication Kinetics

HEK293 T cells and TZM-bl HeLa indicator cells were grown in DMEM, the human T-cell line CEM in RPMI 1640 medium. Both media were supplemented with 10% fetal calf serum, 100 U/ml penicillin and 100 µg/ml streptomycin. For analysis of virus production and infection experiments, 5×10^6^ HEK293 T cells were transiently transfected with 20 µg of the various provirus plasmids, or with pcDNA3 as a control, using PEI (Polyplus) according to the manufacturers instructions, supernatants were harvested 72 h post transfection, filtered through a 0.45 µm pore filter and amounts of capsid protein (CA) were quantified by ELISA using CA-specific antibodies (Polymun) as described [16]. To determine virus infectivity, TZM-bl HeLa indicator cells expressing the firefly luciferase reporter gene under the control of the HIV-1 long terminal repeat promoter were infected with different dilutions of CA-normalized supernatants or incubated with the supernatant of pcDNA3 transfected cells as a control. 48 h after infection, cells were lysed in luciferase lysis buffer (luciferase assay, Promega), and luciferase activity was quantified in a Lumat 9501 luminometer (Berthold) according to the manufacturer’s instructions. To monitor viral replication, equal CA-normalized amounts of wildtype and mutant particles were used to infect 2×10^6^ CEM cells. Supernatant of pcDNA3 transfected cells was used as a control. After 6 h, infected cultures were washed with medium, transferred to new flasks and cultivated for 136 days. At least two times a week half of the culture medium was replaced by fresh medium, and supernatant samples were collected to quantify CA amounts by ELISA to monitor replication and maintenance. Once a week half of the cell culture was replaced by fresh, uninfected cells. At various time points samples of infected cells were collected, pelleted (300 g, 1 min), and genomic DNA was extracted using the QIAamp DNA Mini kit (Qiagen). DNA was PCR amplified with specific primers, purified and subjected to DNA sequencing.

## Supporting Information

Figure S1 **Alignments of two watermarked genes with their non-labeled counterparts.** (A) Alignment of the GFP gene optimized for expression in *H. sapiens* and its counterpart containing the watermark “AEQUOREA VICTORIA.”. Substitutions necessary for watermark integration are highlighted. Only amino acids with 4 or 6 alternative codons were used for embedding the binary message. (B) Alignment of the GFP gene optimized for expression in *H. sapiens* and its counterpart containing the watermark “GREEN FLUORESCENT PROTEIN GENEART 2008”. Substitutions necessary for watermark integration are highlighted. All amino acids with 2, 3, 4 or 6 alternative codons were used for embedding the binary message.
(DOC)

Table S1 **Codon usage tables for the organisms used in this study.** Species-specific codon usage tables (CUT) were used for the optimization of natural genes [1] and embedding the watermark messages into these optimized reading frames. Moitey  =  Percentage of each alternative codon per amino acid. Rank  =  Sorted order of moieties per amino acid starting with the most frequent codon.
(DOC)

File S1 **Sequences of optimized and watermarked genes used in the study.**
(DOC)
