# Supplementary material for: Embedding Permanent Watermarks in Synthetic Genes
Source: PLoS One. 2012 Aug 8;7(8):e42465. doi: 10.1371/journal.pone.0042465 (PMC3414517; doi:10.1371/journal.pone.0042465)
Supplement: Figure S1 — Alignments of two watermarked genes with their non-labeled counterparts. (A) Alignment of the GFP gene optimized for expression in H. sapiens and its counterpart containing the watermark “AEQUOREA VICTORIA.”. Substitutions necessary for watermark integration are highlighted. Only amino acids with 4 or 6 alternative codons were used for embedding the binary message. (B) Alignment of the GFP gene optimized for expression in H. sapiens and its counterpart containing the watermark “GREEN FLUORESCENT PROTEIN GENEART 2008”. Substitutions necessary for watermark integration are highlighted. All amino acids with 2, 3, 4 or 6 alternative codons were used for embedding the binary message. (DOC) [file pone.0042465.s001.doc]

**(A)** Alignment of the GFP gene optimized for expression in *H. sapiens* and its counterpart containing the watermark "AEQUOREA VICTORIA.". Substitutions necessary for watermark integration are highlighted. Only amino acids with 4 or 6 alternative codons were used for embedding the binary message.

**(B)** Alignment of the GFP gene optimized for expression in *H. sapiens* and its counterpart containing the watermark "GREEN FLUORESCENT PROTEIN GENEART 2008". Substitutions necessary for watermark integration are highlighted. All amino acids with 2, 3, 4 or 6 alternative codons were used for embedding the binary message.
