# Supplementary material for: Embedding Permanent Watermarks in Synthetic Genes
Source: PLoS One. 2012 Aug 8;7(8):e42465. doi: 10.1371/journal.pone.0042465 (PMC3414517; doi:10.1371/journal.pone.0042465)
Supplement: Table S1 — Codon usage tables for the organisms used in this study. Species-specific codon usage tables (CUT) were used for the optimization of natural genes [1] and embedding the watermark messages into these optimized reading frames. Moitey = Percentage of each alternative codon per amino acid. Rank = Sorted order of moieties per amino acid starting with the most frequent codon. (DOC) [file pone.0042465.s002.doc]

|  |  |  | ***E. coli*** | |  | ***S. cerevisiae*** | |  | ***A. thaliana*** | |  | ***H. sapiens*** | |
| --- | --- | --- | --- | --- | --- | --- | --- | --- | --- | --- | --- | --- | --- |
| **AA** | **Codon** |  | **Moiety** | **Rank** |  | **Moiety** | **Rank** |  | **Moiety** | **Rank** |  | **Moiety** | **Rank** |
| **A** | **GCA** |  | 22.1% | 3 |  | 28.8% | 2 |  | 26.9% | 2 |  | 22.8% | 3 |
| **A** | **GCC** |  | 26.9% | 2 |  | 22.4% | 3 |  | 15.8% | 3 |  | 40.0% | 1 |
| **A** | **GCG** |  | 34.5% | 1 |  | 11.0% | 4 |  | 13.8% | 4 |  | 10.6% | 4 |
| **A** | **GCT** |  | 16.5% | 4 |  | 37.7% | 1 |  | 43.5% | 1 |  | 26.6% | 2 |
| **C** | **TGC** |  | 55.2% | 1 |  | 37.2% | 2 |  | 40.7% | 2 |  | 54.3% | 1 |
| **C** | **TGT** |  | 44.8% | 2 |  | 62.8% | 1 |  | 59.3% | 1 |  | 45.7% | 2 |
| **D** | **GAC** |  | 36.9% | 2 |  | 34.9% | 2 |  | 32.0% | 2 |  | 53.5% | 1 |
| **D** | **GAT** |  | 63.1% | 1 |  | 65.1% | 1 |  | 68.0% | 1 |  | 46.5% | 2 |
| **E** | **GAA** |  | 67.8% | 1 |  | 70.4% | 1 |  | 51.6% | 1 |  | 42.3% | 2 |
| **E** | **GAG** |  | 32.2% | 2 |  | 29.6% | 2 |  | 48.4% | 2 |  | 57.7% | 1 |
| **F** | **TTC** |  | 41.7% | 2 |  | 41.3% | 2 |  | 48.7% | 2 |  | 53.6% | 1 |
| **F** | **TTT** |  | 58.3% | 1 |  | 58.7% | 1 |  | 51.3% | 1 |  | 46.4% | 2 |
| **G** | **GGA** |  | 12.2% | 4 |  | 21.5% | 2 |  | 36.8% | 1 |  | 25.0% | 2 |
| **G** | **GGC** |  | 38.5% | 1 |  | 19.4% | 3 |  | 14.0% | 4 |  | 33.6% | 1 |
| **G** | **GGG** |  | 16.2% | 3 |  | 11.9% | 4 |  | 15.5% | 3 |  | 25.0% | 3 |
| **G** | **GGT** |  | 33.2% | 2 |  | 47.2% | 1 |  | 33.7% | 2 |  | 16.4% | 4 |
| **H** | **CAC** |  | 42.3% | 2 |  | 36.4% | 2 |  | 38.7% | 2 |  | 58.1% | 1 |
| **H** | **CAT** |  | 57.7% | 1 |  | 63.6% | 1 |  | 61.3% | 1 |  | 41.9% | 2 |
| **I** | **ATA** |  | 9.3% | 3 |  | 27.3% | 2 |  | 24.0% | 3 |  | 16.9% | 3 |
| **I** | **ATC** |  | 40.4% | 2 |  | 26.4% | 3 |  | 35.2% | 2 |  | 47.0% | 1 |
| **I** | **ATT** |  | 50.3% | 1 |  | 46.2% | 1 |  | 40.9% | 1 |  | 36.1% | 2 |
| **K** | **AAA** |  | 75.6% | 1 |  | 57.6% | 1 |  | 48.5% | 2 |  | 43.3% | 2 |
| **K** | **AAG** |  | 24.4% | 2 |  | 42.4% | 2 |  | 51.5% | 1 |  | 56.7% | 1 |
| **L** | **CTA** |  | 3.8% | 6 |  | 14.1% | 3 |  | 10.6% | 5 |  | 7.1% | 6 |
| **L** | **CTC** |  | 10.1% | 5 |  | 5.7% | 6 |  | 17.2% | 3 |  | 19.6% | 2 |
| **L** | **CTG** |  | 49.3% | 1 |  | 11.1% | 5 |  | 10.5% | 6 |  | 39.5% | 1 |
| **L** | **CTT** |  | 11.0% | 4 |  | 12.9% | 4 |  | 25.8% | 1 |  | 13.2% | 3 |
| **L** | **TTA** |  | 13.3% | 2 |  | 27.6% | 2 |  | 13.6% | 4 |  | 7.7% | 5 |
| **L** | **TTG** |  | 12.5% | 3 |  | 28.6% | 1 |  | 22.4% | 2 |  | 12.9% | 4 |
| **M** | **ATG** |  | 100.0% |  |  | 100.0% |  |  | 100.0% |  |  | 100.0% |  |
| **N** | **AAC** |  | 53.1% | 1 |  | 41.0% | 2 |  | 48.4% | 2 |  | 52.9% | 1 |
| **N** | **AAT** |  | 46.9% | 2 |  | 59.0% | 1 |  | 51.6% | 1 |  | 47.1% | 2 |
| **P** | **CCA** |  | 19.3% | 2 |  | 41.7% | 1 |  | 33.1% | 2 |  | 27.7% | 3 |
| **P** | **CCC** |  | 12.8% | 4 |  | 15.5% | 3 |  | 10.9% | 4 |  | 32.4% | 1 |
| **P** | **CCG** |  | 51.4% | 1 |  | 12.1% | 4 |  | 17.7% | 3 |  | 11.3% | 4 |
| **P** | **CCT** |  | 16.5% | 3 |  | 30.8% | 2 |  | 38.4% | 1 |  | 28.6% | 2 |
| **Q** | **CAA** |  | 33.3% | 2 |  | 69.3% | 1 |  | 56.1% | 1 |  | 26.5% | 2 |
| **Q** | **CAG** |  | 66.7% | 1 |  | 30.7% | 2 |  | 43.9% | 2 |  | 73.5% | 1 |
| **R** | **AGA** |  | 5.2% | 5 |  | 48.2% | 1 |  | 35.2% | 1 |  | 21.5% | 1 |
| **R** | **AGG** |  | 3.2% | 6 |  | 20.8% | 2 |  | 20.4% | 2 |  | 21.2% | 2 |
| **R** | **CGA** |  | 6.8% | 4 |  | 6.8% | 4 |  | 11.7% | 4 |  | 10.9% | 5 |
| **R** | **CGC** |  | 37.3% | 1 |  | 5.9% | 5 |  | 7.0% | 6 |  | 18.4% | 4 |
| **R** | **CGG** |  | 11.1% | 3 |  | 3.8% | 6 |  | 9.1% | 5 |  | 20.1% | 3 |
| **R** | **CGT** |  | 36.3% | 2 |  | 14.5% | 3 |  | 16.7% | 3 |  | 7.9% | 6 |
| **S** | **AGC** |  | 26.7% | 1 |  | 11.0% | 5 |  | 12.7% | 4 |  | 24.0% | 1 |
| **S** | **AGT** |  | 15.7% | 2 |  | 16.0% | 3 |  | 15.7% | 3 |  | 14.9% | 5 |
| **S** | **TCA** |  | 13.5% | 6 |  | 21.0% | 2 |  | 20.5% | 2 |  | 15.0% | 4 |
| **S** | **TCC** |  | 14.9% | 3 |  | 16.0% | 4 |  | 12.5% | 5 |  | 21.9% | 2 |
| **S** | **TCG** |  | 14.7% | 4 |  | 9.7% | 6 |  | 10.4% | 6 |  | 5.4% | 6 |
| **S** | **TCT** |  | 14.5% | 5 |  | 26.4% | 1 |  | 28.2% | 1 |  | 18.8% | 3 |
| **T** | **ACA** |  | 14.7% | 4 |  | 30.3% | 2 |  | 30.7% | 2 |  | 28.4% | 2 |
| **T** | **ACC** |  | 41.5% | 1 |  | 21.6% | 3 |  | 20.1% | 3 |  | 35.5% | 1 |
| **T** | **ACG** |  | 27.3% | 2 |  | 13.6% | 4 |  | 15.0% | 4 |  | 11.5% | 4 |
| **T** | **ACT** |  | 16.5% | 3 |  | 34.5% | 1 |  | 34.2% | 1 |  | 24.6% | 3 |
| **V** | **GTA** |  | 15.6% | 4 |  | 20.9% | 2 |  | 14.7% | 4 |  | 11.7% | 4 |
| **V** | **GTC** |  | 21.1% | 3 |  | 20.9% | 3 |  | 19.0% | 3 |  | 23.9% | 2 |
| **V** | **GTG** |  | 37.4% | 1 |  | 19.1% | 4 |  | 25.9% | 2 |  | 46.3% | 1 |
| **V** | **GTT** |  | 25.9% | 2 |  | 39.1% | 1 |  | 40.4% | 1 |  | 18.1% | 3 |
| **W** | **TGG** |  | 100.0% |  |  | 100.0% |  |  | 100.0% |  |  | 100.0% |  |
| **Y** | **TAC** |  | 42.7% | 2 |  | 44.0% | 2 |  | 48.4% | 2 |  | 55.6% | 1 |
| **Y** | **TAT** |  | 57.3% | 1 |  | 56.0% | 1 |  | 51.6% | 1 |  | 44.4% | 2 |
| **Stop** | **TAA** |  | 58.8% | 1 |  | 47.8% | 1 |  | 34.6% | 2 |  | 29.4% | 2 |
| **Stop** | **TAG** |  | 8.8% | 3 |  | 21.7% | 3 |  | 19.2% | 3 |  | 23.5% | 3 |
| **Stop** | **TGA** |  | 32.4% | 2 |  | 30.4% | 2 |  | 46.2% | 1 |  | 47.1% | 1 |
