# Supplementary material for: Embedding Permanent Watermarks in Synthetic Genes
Source: PLoS One. 2012 Aug 8;7(8):e42465. doi: 10.1371/journal.pone.0042465 (PMC3414517; doi:10.1371/journal.pone.0042465)
Supplement: File S1 — Sequences of optimized and watermarked genes used in the study. (DOC) [file pone.0042465.s003.doc]

>GST-T7RNAP(opt)

ATGTCCCCTATACTAGGTTATTGGAAAATTAAGGGCCTTGTGCAACCCACTCGACTTCTTTTGGAATATCTTGAAGAAAAATATGAAGAGCATTTGTATG

AGCGCGATGAAGGTGATAAATGGCGAAACAAAAAGTTTGAATTGGGTTTGGAGTTTCCCAATCTTCCTTATTATATTGATGGTGATGTTAAATTAACACA

GTCTATGGCCATCATACGTTATATAGCTGACAAGCACAACATGTTGGGTGGTTGTCCAAAAGAGCGTGCAGAGATTTCAATGCTTGAAGGAGCGGTTTTG

GATATTAGATACGGTGTTTCGAGAATTGCATATAGTAAAGACTTTGAAACTCTCAAAGTTGATTTTCTTAGCAAGCTACCTGAAATGCTGAAAATGTTCG

AAGATCGTTTATGTCATAAAACATATTTAAATGGTGATCATGTAACCCATCCTGACTTCATGTTGTATGACGCTCTTGATGTTGTTTTATACATGGACCC

AATGTGCCTGGATGCGTTCCCAAAATTAGTTTGTTTTAAAAAACGTATTGAAGCTATCCCACAAATTGATAAGTACTTGAAATCCAGCAAGTATATAGCA

TGGCCTTTGCAGGGCTGGCAAGCCACGTTTGGTGGTGGCGACCATCCTCCAAAATCGGATCTGGTTCCGCGTGGATCCCCGGAATTCGAAGGCGGCGGTA

GCGAAGGTGGCGGCTCTGAAGGTGGTGGCAGCGAAGGCGGTGGCAGCATGAACACCATTAACATCGCGAAAAACGATTTCAGCGATATTGAACTGGCCGC

GATTCCGTTTAACACCCTGGCCGATCATTATGGCGAACGTCTGGCCCGTGAACAGCTGGCCCTGGAACATGAAAGCTATGAAATGGGCGAAGCGCGTTTT

CGTAAAATGTTTGAACGTCAGCTGAAAGCGGGCGAAGTGGCGGATAACGCAGCGGCGAAACCGCTGATTACCACCCTGCTGCCGAAAATGATTGCGCGTA

TCAACGATTGGTTCGAAGAAGTGAAAGCGAAACGCGGCAAACGTCCGACCGCGTTTCAGTTTCTGCAGGAAATTAAACCGGAAGCGGTGGCGTATATTAC

CATTAAAACCACCCTGGCCTGCCTGACCAGCGCGGATAACACCACCGTGCAGGCGGTTGCGAGCGCGATTGGCCGTGCGATTGAAGATGAAGCGCGCTTT

GGCCGTATTCGTGATCTGGAAGCGAAACATTTCAAAAAAAACGTGGAAGAACAGCTGAACAAACGTGTGGGCCACGTGTATAAAAAAGCGTTTATGCAGG

TGGTGGAAGCGGATATGCTGTCTAAAGGCCTGCTGGGCGGTGAAGCGTGGAGCAGCTGGCATAAAGAAGATAGCATTCACGTTGGCGTGCGTTGCATTGA

AATGCTGATTGAAAGCACCGGCATGGTGAGCCTGCATCGTCAGAACGCGGGCGTGGTGGGCCAGGATAGCGAAACCATCGAACTGGCCCCGGAATATGCG

GAAGCGATTGCGACCCGTGCGGGTGCGCTGGCCGGTATTAGCCCGATGTTTCAGCCGTGTGTGGTGCCGCCGAAACCGTGGACCGGCATTACCGGCGGTG

GCTATTGGGCGAACGGCCGTCGTCCGCTGGCCCTGGTGCGTACCCATAGCAAAAAAGCGCTGATGCGTTATGAAGATGTGTATATGCCGGAAGTGTATAA

AGCGATTAACATTGCGCAGAACACCGCGTGGAAAATCAACAAAAAAGTGCTGGCCGTGGCGAACGTGATTACCAAATGGAAACATTGCCCGGTGGAAGAT

ATTCCGGCGATTGAACGTGAAGAACTGCCGATGAAACCGGAAGATATCGATATGAATCCGGAAGCGCTGACCGCCTGGAAACGTGCGGCAGCGGCGGTGT

ATCGTAAAGATAAAGCGCGTAAAAGCCGTCGTATTAGCCTGGAATTTATGCTGGAACAGGCGAACAAATTTGCGAACCATAAAGCGATTTGGTTCCCGTA

TAACATGGATTGGCGTGGCCGTGTGTATGCGGTGAGCATGTTTAATCCGCAGGGCAACGATATGACCAAAGGTCTGCTGACCCTGGCCAAAGGCAAACCG

ATTGGCAAAGAAGGCTATTACTGGCTGAAAATTCATGGCGCGAACTGCGCGGGTGTGGATAAAGTGCCGTTTCCGGAACGCATTAAATTCATCGAAGAAA

ACCACGAAAACATTATGGCGTGCGCGAAAAGCCCGCTGGAAAATACCTGGTGGGCGGAACAGGATAGCCCGTTTTGCTTTCTGGCCTTTTGCTTTGAATA

TGCGGGCGTGCAGCATCATGGCCTGAGCTATAACTGCAGCCTGCCGCTGGCCTTTGATGGCAGCTGCAGCGGCATTCAGCATTTTAGCGCGATGCTGCGT

GATGAAGTGGGCGGTCGTGCGGTGAATCTGCTGCCGAGCGAAACCGTGCAGGATATTTATGGCATCGTGGCGAAAAAAGTGAACGAAATTCTGCAGGCGG

ATGCGATTAACGGCACCGATAACGAAGTGGTGACCGTGACCGATGAAAACACCGGCGAAATTAGCGAAAAAGTGAAACTGGGCACCAAAGCGCTGGCCGG

CCAATGGCTGGCCTATGGCGTGACCCGTAGCGTGACCAAACGTAGCGTGATGACCCTGGCCTATGGTAGCAAAGAATTTGGCTTTCGTCAGCAGGTGCTG

GAAGATACCATTCAGCCGGCGATTGATAGCGGCAAAGGCCTGATGTTTACCCAGCCGAACCAGGCAGCGGGCTATATGGCGAAACTGATTTGGGAAAGCG

TGAGCGTGACCGTGGTTGCGGCGGTGGAAGCGATGAATTGGCTGAAAAGCGCGGCGAAACTGCTGGCCGCGGAAGTGAAAGATAAAAAAACCGGTGAAAT

TCTGCGTAAACGTTGCGCGGTGCATTGGGTGACCCCGGATGGCTTTCCGGTGTGGCAGGAATATAAAAAACCGATTCAGACCCGTCTGAACCTGATGTTT

CTGGGCCAGTTTCGTCTGCAGCCGACCATTAACACCAACAAAGATAGCGAAATCGATGCGCATAAACAGGAAAGCGGCATTGCGCCGAACTTTGTGCATA

GCCAGGATGGCAGCCATCTGCGTAAAACCGTGGTGTGGGCGCATGAAAAATATGGCATCGAAAGCTTCGCGCTGATTCATGATAGCTTTGGCACCATTCC

GGCGGATGCGGCGAACCTGTTTAAAGCGGTGCGTGAAACCATGGTGGATACCTACGAAAGCTGCGATGTGCTGGCCGATTTTTATGATCAGTTTGCGGAT

CAGCTGCATGAAAGCCAGCTGGATAAAATGCCGGCGCTGCCGGCGAAAGGCAACCTGAACCTGCGTGATATTCTGGAAAGCGATTTTGCGTTTGCATGAT

AA

>GST-T7RNAP(msg) "GENEART AG, GERMANY / THE GENE OF YOUR CHOICE / MARCH 19TH 2008 / WAGNER & LISS...."

ATGAGCCCAATATTAGGCTATTGGAAAATTAAGGGCCTGGTGCAACCAACGCGCTTACTGCTGGAATATTTAGAAGAAAAATATGAAGAGCATCTGTATG

AGCGCGATGAAGGCGATAAATGGCGTAACAAAAAGTTTGAACTGGGTTTAGAGTTTCCGAATTTACCGTATTATATTGATGGCGATGTTAAATTAACGCA

GAGTATGGCGATCATACGCTATATAGCGGACAAGCACAACATGTTAGGTGGCTGTCCAAAAGAGCGCGCGGAGATTAGTATGCTGGAAGGTGCCGTTTTA

GATATTCGTTACGGTGTTAGTCGCATTGCCTATAGTAAAGACTTTGAAACGTTAAAAGTGGATTTTCTGAGTAAGTTACCGGAAATGCTGAAAATGTTCG

AAGATCGCTTATGTCATAAAACGTATCTGAATGGCGATCATGTTACGCATCCAGACTTCATGTTATATGACGCCTTAGATGTTGTTCTGTACATGGACCC

AATGTGCTTAGATGCGTTCCCGAAACTGGTGTGTTTTAAAAAACGTATTGAAGCCATCCCGCAAATTGATAAGTACTTAAAAAGCAGCAAGTATATAGCG

TGGCCATTACAGGGCTGGCAAGCCACCTTTGGTGGCGGCGACCATCCACCGAAAAGCGATTTAGTTCCACGTGGCAGCCCAGAATTCGAAGGCGGGGGCA

GTGAAGGCGGCGGCAGTGAAGGTGGCGGTAGTGAAGGTGGTGGTAGTATGAACACGATTAACATCGCCAAAAACGATTTCAGCGATATTGAACTGGCGGC

GATTCCATTTAACACGTTAGCCGATCATTATGGTGAACGTCTGGCGCGTGAACAGCTGGCCTTAGAACATGAAAGCTATGAAATGGGTGAAGCGCGTTTT

CGTAAAATGTTTGAACGTCAGCTGAAAGCCGGTGAAGTGGCCGATAACGCAGCCGCCAAACCATTAATTACGACGCTGTTACCAAAAATGATTGCGCGCA

TCAACGATTGGTTCGAAGAAGTGAAAGCGAAACGTGGTAAACGCCCAACCGCGTTTCAGTTTTTACAGGAAATTAAACCGGAAGCGGTGGCCTATATTAC

CATTAAAACGACGCTGGCCTGCCTGACGAGTGCCGATAACACGACGGTTCAGGCGGTTGCGAGCGCGATTGGCCGCGCCATTGAAGATGAAGCCCGCTTT

GGCCGTATTCGTGATTTAGAAGCCAAACATTTCAAAAAAAACGTTGAAGAACAGTTAAACAAACGTGTGGGCCACGTGTATAAAAAAGCCTTTATGCAGG

TTGTGGAAGCGGATATGTTAAGCAAAGGCCTGCTGGGCGGCGAAGCCTGGAGCAGTTGGCATAAAGAAGATAGCATTCACGTCGGCGTTCGTTGCATTGA

AATGCTGATTGAAAGTACGGGTATGGTTAGTTTACATCGTCAGAACGCGGGTGTTGTTGGCCAGGATAGCGAAACCATCGAATTAGCGCCAGAATATGCC

GAAGCCATTGCGACGCGCGCGGGCGCGCTGGCCGGCATTAGTCCAATGTTTCAGCCGTGTGTGGTTCCACCAAAACCGTGGACCGGCATTACGGGTGGCG

GTTATTGGGCGAACGGTCGTCGTCCATTAGCCTTAGTTCGCACCCATAGCAAAAAAGCGTTAATGCGTTATGAAGATGTTTATATGCCAGAAGTTTATAA

AGCCATTAACATTGCGCAGAACACGGCGTGGAAAATCAACAAAAAAGTGTTAGCGGTGGCCAACGTTATTACGAAATGGAAACATTGCCCAGTGGAAGAT

ATTCCGGCGATTGAACGTGAAGAATTACCGATGAAACCCGAAGATATCGATATGAATCCGGAAGCCTTAACGGCGTGGAAACGCGCAGCCGCGGCCGTTT

ATCGTAAAGATAAAGCCCGTAAAAGTCGTCGTATTAGTTTAGAATTTATGCTGGAACAGGCCAACAAATTTGCCAACCATAAAGCCATTTGGTTCCCGTA

TAACATGGATTGGCGTGGCCGCGTTTATGCCGTGAGCATGTTTAATCCGCAGGGTAACGATATGACCAAAGGTTTACTGACGCTGGCCAAAGGTAAACCA

ATTGGTAAAGAAGGTTATTACTGGTTAAAAATTCATGGTGCCAACTGCGCCGGTGTGGATAAAGTTCCATTTCCGGAACGTATTAAATTCATCGAAGAAA

ACCACGAAAACATTATGGCCTGCGCGAAAAGTCCATTAGAAAATACGTGGTGGGCCGAACAGGATAGCCCATTTTGCTTTTTAGCCTTTTGCTTTGAATA

TGCCGGTGTGCAGCATCATGGCTTAAGTTATAACTGCAGTTTACCATTAGCCTTTGATGGTAGTTGCAGTGGTATTCAGCATTTTAGCGCGATGCTGCGC

GATGAAGTTGGTGGTCGTGCCGTTAATCTGCTGCCAAGCGAAACCGTGCAGGATATTTATGGCATCGTTGCCAAAAAAGTTAACGAAATTTTACAGGCGG

ATGCGATTAACGGTACGGATAACGAAGTGGTGACCGTGACGGATGAAAACACCGGCGAAATTAGCGAAAAAGTTAAACTGGGTACGAAAGCGTTAGCGGG

CCAATGGCTGGCCTATGGTGTGACGCGTAGTGTTACGAAACGTAGTGTTATGACGTTAGCGTATGGCAGTAAAGAATTTGGTTTTCGTCAGCAGGTTTTA

GAAGATACGATTCAGCCAGCGATTGATAGTGGCAAAGGCTTAATGTTTACGCAGCCGAACCAGGCCGCGGGTTATATGGCCAAACTGATTTGGGAAAGCG

TGAGTGTTACCGTGGTGGCGGCCGTTGAAGCGATGAATTGGCTGAAAAGTGCCGCGAAACTGCTGGCCGCCGAAGTTAAAGATAAAAAAACCGGCGAAAT

TCTGCGTAAACGTTGCGCCGTGCATTGGGTGACCCCAGATGGTTTTCCAGTGTGGCAGGAATATAAAAAACCGATTCAGACCCGTCTGAACCTGATGTTT

CTGGGCCAGTTTCGTCTGCAGCCGACCATTAACACCAACAAAGATAGCGAAATCGATGCGCATAAACAGGAAAGCGGCATTGCGCCGAACTTTGTGCATA

GCCAGGATGGCAGCCATCTGCGTAAAACCGTGGTGTGGGCGCATGAAAAATATGGCATCGAAAGTTTCGCGCTGATTCATGATAGCTTTGGCACCATTCC

GGCGGATGCGGCGAACCTGTTTAAAGCGGTGCGTGAAACCATGGTGGATACCTACGAAAGCTGCGATGTGCTGGCCGATTTTTATGATCAGTTTGCGGAT

CAGCTGCATGAAAGCCAGCTGGATAAAATGCCGGCGCTGCCGGCGAAAGGCAACCTGAACCTGCGTGATATTCTGGAAAGCGATTTTGCGTTTGCATGAT

AA

>EMG1(opt)

ATGGCTGCTCCTAGCGACGGCTTCAAGCCCCGGGAGCGGAGCGGCGGAGAGCAGGCCCAGGACTGGGACGCCCTGCCCCCCAAGCGGCCTAGACTGGGAG

CCGGCAACAAGATCGGCGGCAGGCGGCTGATCGTGGTGCTGGAAGGCGCCAGCCTGGAAACCGTGAAAGTGGGCAAGACCTACGAGCTGCTGAACTGCGA

CAAGCACAAGAGCATCCTGCTGAAGAACGGCCGGGACCCCGGCGAGGCCAGGCCCGACATCACCCACCAGAGCCTGCTGATGCTCATGGATTCCCCCCTG

AACAGAGCCGGCCTGCTGCAGGTGTACATCCACACCCAGAAAAACGTGCTGATCGAGGTGAACCCCCAGACCAGAATCCCCCGGACCTTCGACCGGTTCT

GCGGCCTGATGGTCCAGCTGCTCCATAAGCTGTCCGTGAGAGCCGCCGACGGCCCCCAGAAACTGCTGAAGGTGATCAAGAACCCCGTGAGCGACCACTT

CCCCGTGGGCTGCATGAAAGTGGGGACCAGCTTCAGCATCCCCGTGGTGTCCGACGTGCGGGAGCTGGTGCCCAGCAGCGACCCCATCGTGTTCGTGGTG

GGCGCCTTCGCCCACGGCAAGGTGTCCGTGGAGTACACCGAGAAGATGGTGTCCATCAGCAACTACCCCCTGTCTGCCGCCCTGACCTGCGCCAAGCTGA

CCACCGCCTTCGAGGAAGTGTGGGGCGTGATCCACCACCACCACCACCACTGATAA

>EMG1(msg) "GENEART AG PAT US1234567"

ATGGCCGCTCCTAGCGACGGCTTCAAGCCCAGAGAGCGCTCCGGCGGAGAGCAGGCCCAGGACTGGGACGCCCTCCCCCCCAAGAGACCTAGACTCGGAG

CCGGAAACAAGATCGGCGGCAGGAGGCTCATCGTCGTGCTGGAAGGCGCTTCCCTGGAAACAGTGAAAGTGGGAAAGACCTACGAGTTGCTCAACTGCGA

CAAGCACAAGTCCATCCTCCTCAAGAACGGAAGGGACCCTGGCGAGGCTAGGCCTGACATCACACACCAGAGCCTGCTCATGCTCATGGATAGCCCCCTG

AACAGGGCTGGACTCCTCCAGGTCTACATCCACACCCAGAAAAACGTGCTCATCGAGGTCAACCCTCAGACAAGAATCCCTAGGACATTCGACAGGTTCT

GCGGCCTGATGGTGCAGCTCCTGCATAAGCTCTCCGTCAGGGCTGCTGACGGACCTCAGAAACTGCTGAAGGTCATCAAGAACCCCGTCAGCGACCACTT

CCCCGTGGGATGCATGAAAGTCGGCACCTCATTCAGCATCCCTGTCGTCAGCGACGTCAGAGAGTTGGTCCCCTCCTCCGACCCCATCGTCTTCGTCGTG

GGCGCTTTCGCCCACGGAAAGGTGTCCGTCGAGTACACAGAGAAGATGGTGTCCATCAGCAACTACCCTCTGTCCGCCGCTCTGACCTGCGCTAAGCTCA

CCACAGCCTTCGAGGAAGTGTGGGGCGTGATCCACCACCACCACCACCACTGATAA

>EMG1(enc) ":JQWF&G%DY%$4Y#'XE%87G;K"

ATGGCTGCCCCCTCCGACGGCTTCAAGCCTAGAGAGAGGAGCGGAGGGGAGCAGGCTCAGGACTGGGACGCCCTGCCTCCTAAGAGGCCCAGACTGGGAG

CCGGCAACAAGATCGGCGGCAGGAGGCTGATCGTTGTCCTCGAAGGAGCTAGCCTGGAAACAGTGAAAGTCGGAAAGACCTACGAGCTGCTGAACTGCGA

CAAGCACAAGTCCATCCTCCTCAAGAACGGCAGGGACCCCGGCGAGGCTAGGCCCGACATCACACACCAGTCCCTGCTGATGCTGATGGATTCCCCTCTG

AACAGGGCTGGACTGCTCCAGGTGTACATCCACACACAGAAAAACGTCCTCATCGAGGTTAACCCTCAGACAAGGATCCCCAGGACCTTCGACAGGTTCT

GCGGACTGATGGTGCAGCTGCTCCATAAGCTCAGCGTCAGGGCTGCTGACGGCCCCCAGAAACTCCTCAAAGTCATCAAGAACCCCGTTAGCGACCACTT

CCCCGTGGGCTGCATGAAAGTCGGAACAAGCTTCTCCATCCCTGTTGTCAGCGACGTCAGGGAGTTGGTGCCTAGCTCCGACCCCATCGTGTTCGTCGTC

GGAGCTTTCGCCCACGGAAAAGTTAGCGTGGAGTACACCGAGAAGATGGTCTCCATCAGCAACTACCCCCTGTCCGCAGCCCTCACCTGCGCCAAGCTGA

CAACCGCTTTCGAGGAAGTGTGGGGCGTGATCCACCACCACCACCACCACTGATAA

>eGFP(opt S.c.)

ATGGTTTCTAAAGGTGAAGAATTGTTTACTGGTGTTGTTCCAATTTTGGTTGAATTGGATGGTGATGTTAATGGTCATAAGTTTTCTGTTTCTGGTGAAG

GTGAAGGTGATGCTACTTATGGTAAATTGACTTTGAAGTTCATTTGTACTACTGGTAAATTGCCAGTTCCATGGCCAACTTTGGTTACTACTTTGACTTA

TGGTGTTCAATGTTTTTCAAGATACCCAGATCACATGAAGCAACATGATTTTTTCAAATCTGCTATGCCAGAAGGTTACGTTCAAGAAAGAACTATTTTC

TTCAAGGATGATGGTAATTACAAAACTAGAGCTGAAGTTAAATTTGAAGGTGATACTTTGGTTAACAGAATTGAATTGAAGGGTATTGATTTCAAAGAAG

ATGGTAACATTTTGGGTCATAAGTTGGAATACAACTACAATTCTCATAACGTTTACATTATGGCTGATAAGCAAAAGAATGGTATTAAGGTTAACTTCAA

GATTAGACATAACATTGAAGATGGTTCTGTTCAATTGGCTGATCATTACCAACAAAATACTCCAATTGGTGATGGTCCAGTTTTGTTGCCAGATAATCAT

TATTTGTCTACTCAATCTGCTTTGTCTAAAGATCCAAACGAGAAGAGGGATCACATGGTTTTGTTGGAATTTGTTACTGCTGCTGGTATTACTTTGGGTA

TGGATGAATTGTATAAGCATCATCACCATCACCATTAGTAA

>eGFP(msg S.c.) "AEQUOREA VICTORIA."

ATGGTTTCAAAAGGAGAAGAATTATTTACAGGTGTTGTACCTATTTTGGTAGAATTGGATGGTGATGTTAATGGACATAAGTTTTCAGTATCTGGTGAAG

GTGAAGGAGATGCTACATATGGTAAATTGACATTGAAGTTCATTTGTACTACTGGTAAATTGCCAGTACCTTGGCCAACATTGGTAACAACTTTAACTTA

TGGTGTACAATGTTTTTCACGATACCCTGATCACATGAAGCAACATGATTTTTTCAAATCTGCAATGCCTGAAGGATACGTACAAGAAAGGACAATTTTC

TTCAAGGATGATGGTAATTACAAAACTAGGGCTGAAGTTAAATTTGAAGGAGATACTTTAGTTAACAGGATTGAATTAAAGGGTATTGATTTCAAAGAAG

ATGGTAACATTTTAGGACATAAGTTAGAATACAACTACAATTCTCATAACGTTTACATTATGGCTGATAAGCAAAAGAATGGTATTAAGGTAAACTTCAA

GATTAGACATAACATTGAAGATGGATCAGTTCAATTAGCTGATCATTACCAACAAAATACTCCAATTGGTGATGGTCCAGTATTATTGCCTGATAATCAT

TATTTGTCAACTCAATCAGCATTGTCTAAAGATCCTAACGAGAAGAGGGATCACATGGTATTATTGGAATTTGTAACAGCTGCTGGTATTACATTGGGTA

TGGATGAATTGTATAAGCATCATCACCATCACCATTAGTAA

>eGFP(opt A.t.)

ATGGTTTCTAAGGGTGAAGAGTTGTTCACTGGTGTTGTTCCTATCCTTGTGGAGCTTGATGGTGATGTTAACGGACACAAGTTCTCTGTTTCTGGTGAAG

GTGAAGGTGATGCTACTTACGGAAAGCTCACCCTTAAGTTCATCTGCACTACTGGAAAGTTGCCTGTTCCTTGGCCTACTCTTGTTACTACTCTCACCTA

CGGTGTTCAGTGTTTCTCTAGATACCCTGATCACATGAAGCAGCACGATTTCTTCAAGTCTGCTATGCCTGAAGGATACGTGCAAGAGAGAACCATCTTC

TTCAAGGATGATGGAAACTACAAGACTAGAGCTGAGGTTAAGTTCGAGGGTGATACTCTCGTTAACAGGATCGAGCTTAAGGGAATCGATTTCAAAGAGG

ATGGAAACATCCTTGGACATAAGTTGGAGTACAACTACAACTCTCACAACGTGTACATCATGGCTGATAAGCAGAAGAACGGTATCAAGGTTAACTTCAA

GATCAGGCACAACATTGAGGATGGATCTGTTCAGCTTGCTGATCATTACCAACAGAACACCCCTATTGGAGATGGACCTGTTCTTCTCCCTGATAACCAC

TACCTTTCTACTCAGTCTGCTCTCTCTAAGGATCCTAACGAGAAGAGGGATCACATGGTTCTTTTGGAGTTTGTTACTGCTGCTGGAATCACTCTTGGAA

TGGATGAGCTTTACAAGCTCGAGCACCACCATCACCATCACCATCACTAA

>eGFP(msg A.t.) "AEQUOREA VICTORIA."

ATGGTTTCAAAGGGTGAAGAGTTGTTCACAGGAGTTGTGCCAATCCTTGTGGAGCTTGATGGAGATGTTAACGGTCACAAGTTCTCAGTGTCTGGAGAAG

GAGAAGGTGATGCTACATACGGAAAGCTTACACTTAAGTTCATCTGCACTACTGGAAAGCTTCCTGTGCCCTGGCCTACACTTGTGACAACTTTGACTTA

CGGAGTGCAGTGTTTCTCACGATACCCAGATCACATGAAGCAGCACGATTTCTTCAAGTCTGCAATGCCAGAAGGTTACGTGCAAGAGAGGACAATCTTC

TTCAAGGATGATGGAAACTACAAGACTAGGGCTGAGGTTAAGTTCGAGGGTGATACTTTGGTTAACAGGATCGAGTTGAAGGGAATCGATTTCAAAGAGG

ATGGAAACATCTTGGGTCATAAGTTGGAGTACAACTACAACTCTCACAACGTTTACATCATGGCTGATAAGCAGAAGAACGGAATCAAGGTGAACTTCAA

GATCAGACACAACATTGAGGATGGTTCAGTTCAGTTGGCTGATCATTACCAACAGAACACTCCTATTGGAGATGGACCTGTGTTGCTTCCAGATAACCAC

TACCTTTCAACTCAGTCAGCACTTTCTAAGGATCCAAACGAGAAGAGGGATCACATGGTGTTGCTTGAGTTTGTGACAGCTGCTGGAATCACACTTGGAA

TGGATGAGCTTTACAAGCTCGAGCACCACCATCACCATCACCATCACTAA

>eGFP(opt H.s.)

ATGGTGTCCAAGGGCGAGGAACTGTTCACCGGCGTGGTGCCCATCCTGGTGGAGCTGGACGGCGACGTGAACGGCCACAAGTTCAGCGTGTCCGGCGAGG

GCGAAGGCGACGCCACCTACGGCAAGCTGACCCTGAAGTTCATCTGCACCACCGGCAAGCTGCCCGTGCCCTGGCCCACCCTGGTCACCACCCTGACCTA

CGGCGTGCAGTGCTTCAGCAGATACCCCGACCACATGAAGCAGCACGATTTCTTCAAGAGCGCCATGCCCGAGGGCTACGTGCAGGAACGGACCATCTTC

TTCAAGGACGACGGCAACTACAAGACCAGAGCCGAAGTGAAGTTCGAGGGCGACACACTGGTCAACCGGATCGAGCTGAAGGGCATCGACTTCAAAGAGG

ACGGCAATATCCTGGGCCACAAGCTGGAATACAACTACAACAGCCACAACGTGTACATCATGGCCGACAAGCAGAAGAACGGCATCAAAGTCAACTTCAA

GATCCGGCACAACATCGAGGACGGCTCTGTGCAGCTGGCTGACCACTACCAGCAGAACACCCCCATCGGCGACGGCCCCGTGCTGCTGCCCGACAACCAC

TACCTGAGCACCCAGAGCGCCCTGAGCAAGGACCCCAACGAGAAGCGGGACCACATGGTGCTGCTGGAATTCGTGACAGCCGCCGGAATCACCCTGGGCA

TGGACGAGCTGTACAAGCTCGAGCACCACCATCACCATCACCATCACTAA

>eGFP(msg H.s.) "AEQUOREA VICTORIA."

ATGGTGTCCAAGGGAGAGGAACTCTTCACAGGCGTGGTCCCTATCCTGGTCGAGCTGGACGGCGACGTGAACGGACACAAGTTCTCCGTCAGCGGCGAGG

GCGAAGGAGACGCCACATACGGCAAGCTGACACTGAAGTTCATCTGCACCACCGGCAAGCTGCCCGTCCCTTGGCCCACACTGGTCACAACCCTCACCTA

CGGCGTCCAGTGCTTCTCCCGCTACCCTGACCACATGAAGCAGCACGATTTCTTCAAGAGCGCTATGCCTGAGGGATACGTCCAGGAAAGGACAATCTTC

TTCAAGGACGACGGCAACTACAAGACCAGGGCCGAAGTGAAGTTCGAGGGAGACACCCTCGTGAACAGGATCGAGTTGAAGGGCATCGACTTCAAAGAGG

ACGGCAATATCCTCGGACACAAGCTCGAATACAACTACAACAGCCACAACGTGTACATCATGGCCGACAAGCAGAAGAACGGCATCAAAGTCAACTTCAA

GATCAGACACAACATCGAGGACGGATCCGTGCAGCTCGCCGACCACTACCAGCAGAACACCCCCATCGGCGACGGCCCCGTCCTCCTGCCTGACAACCAC

TACCTGTCCACCCAGTCCGCTCTGAGCAAGGACCCTAACGAGAAGAGGGACCACATGGTCCTCCTGGAATTCGTCACAGCCGCCGGCATCACACTGGGCA

TGGACGAGCTGTACAAGCTCGAGCACCACCATCACCATCACCATCACTAA

>eGFP(msg_cut H.s.) "AEQUOREA VICTORIA."

ATGGTGTCCAAGGGAGAGGAACTCTTCACAGGCGTGGTCCCTATCCTGGTCGAGCTGGACGGCGACGTGAACGGACACAAGTTCTCCGTCAGCGGCGAGG

GCGAAGGAGACGCCACATACGGCAAGCTGACACTGAAGTTCATCTGCACCACCGGCAAGCTGCCCGTCCCTTGGCCCACACTGGTCACAACCCTCACCTA

CGGCGTCCAGTGCTTCTCCCGCTACCCTGACCACATGAAGCAGCACGATTTCTTCAAGAGCGCTATGCCTGAGGGATACGTCCAGGAAAGGACAATCTTC

TTCAAGGACGACGGCAACTACAAGACCAGGGCCGAAGTGAAGTTCGAGGGAGACACCCTCGTGAACAGGATCGAGTTGAAGGGCATCGACTTCAAAGAGG

ACGGCAATATCCTCGGACACAAGCTCGAATACAACTACAACAGCCACAACGTGTACATCATGGCCGACAAGCAGAAGAACGGCATCAAAGTCAACTTCAA

GATCAGACACAACATCGAGGACGGATCCGTGCAGCTCGCCGACCACTACCAGCAGAACACCCCCATCGGCGACGGCCCCGTCCTCCTGCCTGACAACCAC

TACCTGTCCACCCAGTCCGCTCTGAGCAAGGACCCTAACGAGAAGAGGGACCACATGGTCCTCCTGGAATTCGTCACAGCCGCCGGCATCACACTGGGCA

TGGACGAGCTGTACAAGCTCGAGCACCACCATCACCATCACCATCACTAACGGCACGCATATGAGCGTAAATGACCCCATTCAAT

>eGFP(msg_enc H.s.) "4JT'T&8F#(NWGTU[FB"

ATGGTCAGCAAGGGAGAGGAACTGTTCACAGGAGTGGTCCCCATCCTCGTGGAGTTGGACGGCGACGTGAACGGACACAAGTTCAGCGTCTCCGGAGAGG

GAGAAGGAGACGCCACCTACGGCAAGCTGACCCTCAAGTTCATCTGCACCACAGGAAAGCTCCCTGTCCCCTGGCCCACACTCGTGACCACACTCACATA

CGGCGTCCAGTGCTTCTCCAGATACCCCGACCACATGAAGCAGCACGATTTCTTCAAGTCCGCTATGCCTGAGGGATACGTCCAGGAAAGAACCATCTTC

TTCAAGGACGACGGAAACTACAAGACAAGAGCTGAAGTCAAGTTCGAGGGAGACACCCTCGTGAACAGAATCGAGCTGAAGGGAATCGACTTCAAAGAGG

ACGGCAATATCCTGGGACACAAGCTGGAATACAACTACAACAGCCACAACGTGTACATCATGGCCGACAAGCAGAAGAACGGAATCAAAGTCAACTTCAA

GATCAGACACAACATCGAGGACGGCAGCGTGCAGCTGGCTGACCACTACCAGCAGAACACCCCTATCGGAGACGGCCCCGTCCTGCTCCCCGACAACCAC

TACCTGAGCACCCAGTCCGCCCTGAGCAAGGACCCTAACGAGAAGAGGGACCACATGGTGCTGCTCGAATTCGTGACAGCTGCTGGCATCACACTGGGCA

TGGACGAGCTGTACAAGCTCGAGCACCACCATCACCATCACCATCACTAA

>eGFP(msg_long H.s.) "GREEN FLUORESCENT PROTEIN GENEART 2008"

ATGGTGTCCAAAGGCGAGGAGCTGTTCACAGGAGTGGTCCCCATTCTCGTGGAACTGGACGGAGATGTGAATGGCCACAAATTCAGCGTGTCCGGAGAAG

GAGAAGGAGATGCCACATATGGCAAGCTCACCCTCAAGTTCATTTGTACCACCGGAAAGCTCCCCGTGCCTTGGCCCACCCTGGTGACCACCCTCACATA

CGGAGTGCAATGTTTCTCCAGATACCCCGATCATATGAAGCAGCACGATTTTTTTAAGAGCGCCATGCCTGAAGGCTATGTGCAGGAAAGAACCATCTTT

TTCAAGGATGACGGAAATTATAAAACAAGGGCTGAAGTGAAGTTTGAAGGAGATACCCTGGTCAATAGAATTGAGTTGAAGGGCATCGACTTCAAGGAAG

ACGGAAATATCCTCGGACACAAACTGGAGTATAACTATAATAGCCACAATGTGTACATCATGGCTGATAAACAAAAAAATGGAATCAAAGTCAACTTCAA

GATCAGGCATAACATTGAGGACGGAAGCGTGCAGCTCGCCGATCATTACCAACAGAACACACCTATTGGAGACGGCCCCGTCCTCCTGCCTGACAACCAT

TACCTCTCCACACAATCCGCTCTCTCCAAAGACCCTAATGAGAAAAGGGACCATATGGTCCTCCTCGAATTCGTCACAGCTGCTGGAATCACCCTCGGAA

TGGATGAGCTGTACAAGCTCGAGCACCACCATCACCATCACCATCACTAA

>HIVgag(opt H.s.)

ATGGGCGCCAGGGCCAGCGTGCTGAGCGGCGGCGAGCTGGACAGGTGGGAGAAGATCAGGCTGAGGCCCGGCGGCAAGAAGAAGTATAAGCTGAAGCACA

TCGTGTGGGCCAGCAGGGAGCTGGAGAGGTTCGCCGTGAACCCCGGCCTGCTGGAGACCAGCGAGGGCTGCAGGCAGATCCTGGGCCAGCTGCAGCCCAG

CCTGCAGACCGGCAGCGAGGAGCTGAGGAGCCTGTACAACACCGTGGCCACCCTGTACTGCGTGCACCAGAGGATCGAGATCAAGGACACCAAGGAGGCC

CTGGACAAGATCGAGGAGGAGCAGAACAAGTCCAAGAAGAAGGCCCAGCAGGCCGCCGCCGACACCGGCCACAGCAGCCAGGTGAGCCAGAACTACCCCA

TCGTGCAGAACATCCAGGGCCAGATGGTGCACCAGGCCATCAGCCCCAGGACCCTGAACGCCTGGGTGAAGGTGGTGGAGGAGAAGGCCTTCAGCCCCGA

GGTGATCCCCATGTTCAGCGCCCTGAGCGAGGGAGCCACCCCCCAGGACCTGAACACCATGCTGAACACCGTGGGCGGCCACCAGGCCGCCATGCAGATG

CTGAAGGAGACCATCAACGAGGAGGCCGCCGAGTGGGACAGGGTGCACCCCGTGCACGCCGGCCCCATCGCCCCCGGCCAGATGAGGGAGCCCCGCGGCA

GCGACATCGCCGGCACCACCAGCACCCTGCAGGAGCAGATCGGCTGGATGACCAACAACCCCCCCATCCCCGTGGGCGAAATCTACAAGAGGTGGATCAT

CCTGGGCCTGAACAAGATCGTGAGGATGTACAGCCCCACCAGCATCCTGGATATCAGGCAGGGCCCCAAAGAGCCCTTCAGGGACTACGTGGACAGGTTC

TACAAGACCCTGCGCGCCGAGCAGGCCAGCCAGGAGGTGAAGAACTGGATGACCGAGACCCTGCTGGTGCAGAACGCCAACCCCGACTGCAAGACCATCC

TGAAGGCCCTGGGACCCGCCGCCACCCTGGAGGAGATGATGACCGCCTGCCAGGGCGTGGGCGGCCCCGGCCACAAGGCCAGGGTGCTGGCCGAGGCCAT

GAGCCAGGTGACCAACACCGCCACCATCATGATGCAGAGGGGCAACTTCAGGAACCAGAGGAAGATGGTGAAGTGCTTCAACTGCGGCAAGGAGGGCCAC

ACCGCCAGGAACTGCCGCGCCCCCAGGAAGAAGGGCTGCTGGAAGTGCGGCAAGGAGGGCCACCAGATGAAGGACTGCACCGAGAGGCAGGCCAACTTCC

TGGGCAAGATCTGGCCCAGCTACAAGGGCAGGCCCGGCAACTTCCTGCAGAGCAGGCCCGAGCCCACCGCCCCCCCCTTCCTGCAGAGCAGGCCCGAGCC

CACCGCCCCCCCCGAGGAGAGCTTCAGGAGCGGCGTGGAGACCACCACCCCTCCTCAGAAGCAGGAGCCCATCGACAAGGAGCTGTACCCCCTGACCAGC

CTGAGGAGCCTGTTCGGCAACGACCCCAGCAGCCAGTGA

>HIVgag(msg H.s.) "GENE DESIGNED BY MARCUS GRAF / GENEART 2008.."

ATGGGCGCTAGGGCCAGCGTGCTGTCCGGAGGCGAGTTGGACAGATGGGAGAAGATCAGACTCAGACCCGGCGGAAAGAAGAAGTATAAGCTGAAGCACA

TCGTCTGGGCTAGCAGGGAGCTGGAGAGGTTCGCTGTCAACCCTGGACTCCTGGAGACATCCGAGGGCTGCAGGCAGATCCTCGGCCAGCTCCAGCCTAG

CCTCCAGACCGGCAGCGAGGAGTTGAGGAGCCTGTACAACACCGTCGCCACACTCTACTGCGTGCACCAGAGAATCGAGATCAAGGACACAAAGGAGGCT

CTGGACAAGATCGAGGAGGAGCAGAACAAGAGCAAGAAGAAGGCCCAGCAGGCCGCTGCCGACACCGGCCACTCCAGCCAGGTCTCCCAGAACTACCCCA

TCGTCCAGAACATCCAGGGCCAGATGGTGCACCAGGCTATCTCCCCCAGGACACTCAACGCTTGGGTCAAGGTCGTCGAGGAGAAGGCTTTCAGCCCTGA

GGTCATCCCTATGTTCAGCGCTCTGAGCGAGGGCGCTACACCCCAGGACCTCAACACAATGCTCAACACAGTCGGAGGCCACCAGGCTGCCATGCAGATG

CTGAAGGAGACAATCAACGAGGAGGCCGCCGAGTGGGACAGGGTCCACCCTGTCCACGCCGGCCCCATCGCTCCTGGCCAGATGAGGGAGCCCAGGGGAT

CCGACATCGCCGGCACCACCTCCACCCTCCAGGAGCAGATCGGCTGGATGACCAACAACCCCCCTATCCCTGTGGGCGAAATCTACAAGAGGTGGATCAT

CCTCGGACTCAACAAGATCGTCAGGATGTACAGCCCTACAAGCATCCTGGATATCAGACAGGGCCCCAAAGAGCCTTTCAGGGACTACGTGGACAGGTTC

TACAAGACCCTCAGGGCTGAGCAGGCTAGCCAGGAGGTGAAGAACTGGATGACAGAGACACTGCTGGTCCAGAACGCTAACCCTGACTGCAAGACAATCC

TCAAGGCTCTCGGACCTGCCGCCACCCTGGAGGAGATGATGACAGCTTGCCAGGGAGTCGGAGGACCCGGACACAAGGCTAGAGTGCTGGCCGAGGCTAT

GTCCCAGGTGACAAACACCGCCACAATCATGATGCAGAGAGGCAACTTCAGAAACCAGAGGAAGATGGTGAAGTGCTTCAACTGCGGAAAGGAGGGACAC

ACCGCTAGAAACTGCCGGGCTCCTAGGAAGAAGGGATGCTGGAAGTGCGGCAAGGAGGGCCACCAGATGAAGGACTGCACCGAGAGGCAGGCTAACTTCC

TGGGAAAGATCTGGCCCAGCTACAAGGGAAGACCTGGAAACTTCCTCCAGTCCAGGCCTGAGCCTACAGCTCCCCCTTTCCTCCAGAGCAGGCCTGAGCC

CACAGCTCCTCCTGAGGAGTCCTTCAGATCCGGAGTCGAGACAACAACCCCCCCTCAGAAGCAGGAGCCTATCGACAAGGAGTTGTACCCTCTCACCAGC

CTGAGGTCCCTCTTCGGCAACGACCCCAGCTCCCAGTGA

>NL4-3(wt)

ACTAGTACCCTTCAGGAACAAATAGGATGGATGACACATAATCCACCTATCCCAGTAGGAGAAATCTATAAAAGATGGATAATCCTGGGATTAAATAAAA

TAGTAAGAATGTATAGCCCTACCAGCATTCTGGACATAAGACAAGGACCAAAGGAACCCTTTAGAGACTATGTAGACCGATTCTATAAAACTCTAAGAGC

CGAGCAAGCTTCACAAGAGGTAAAAAATTGGATGACAGAAACCTTGTTGGTCCAAAATGCGAACCCAGATTGTAAGACTATTTTAAAAGCATTGGGACCA

GGAGCGACACTAGAAGAAATGATGACAGCATGTCAGGGAGTGGGGGGACCCGGCCATAAAGCAAGAGTTTTGGCTGAAGCAATGAGCCAAGTAACAAATC

CAGCTACCATAATGATACAGAAAGGCAATTTTAGGAACCAAAGAAAGACTGTTAAGTGTTTCAATTGTGGCAAAGAAGGGCACATAGCCAAAAATTGCAG

GGCCC

>NL4-3(msg) "[REGENSBURG]"

ACTAGTACCCTTCAGGAACAAATAGGATGGATGACTCATAATCCACCAATCCCAGTAGGGGAAATCTATAAAAGGTGGATAATCTTAGGGTTAAATAAAA

TAGTGAGGATGTATAGTCCTACCAGTATTCTGGACATAAGGCAAGGACCAAAGGAACCCTTTAGAGACTATGTGGACAGGTTCTATAAAACACTGAGAGC

CGAGCAAGCTTCACAAGAGGTAAAAAATTGGATGACAGAAACTTTATTAGTCCAAAATGCGAACCCAGATTGTAAGACAATTTTAAAAGCTTTGGGGCCA

GGGGCAACACTGGAAGAAATGATGACAGCTTGTCAGGGAGTAGGAGGGCCTGGCCATAAAGCTAGAGTGTTGGCAGAAGCAATGAGTCAAGTAACAAATC

CAGCAACTATAATGATACAGAAAGGCAATTTTAGGAACCAAAGAAAGACTGTTAAGTGTTTCAATTGTGGCAAAGAAGGGCACATAGCCAAAAATTGCAG

GGCCC
